# Integrated multi-omics analysis reveals the molecular mechanism of tuber morphogenesis under different planting densities in yam (*Dioscorea opposita* Thunb.)

**DOI:** 10.3389/fpls.2026.1849989

**Published:** 2026-05-19

**Authors:** Peng Wang, Guixiao La, Xiangyang Li, Dandan Dai, Guixia Shi, Yi Wen, Tiegang Yang

**Affiliations:** 1 Institute of Chinese Herbal Medicines, Henan Academy of Agricultural Sciences, Zhengzhou, Henan, China; 2 Key Laboratory for the Protection and Utilization of Chinese Herbal Medicine Resources of Henan Province, Institute of Chinese Herbal Medicines, Henan Academy of Agricultural Sciences, Zhengzhou, Henan, China

**Keywords:** morphological formation, plant hormone signaling pathway, planting density, transcription factors (TFs), yam tubers

## Abstract

Chinese yam (*Dioscorea opposita* Thunb.) is a typical medicinal and edible homologous plant, whose tubers are used for food or medicine. The length and thickness of the tubers directly determine their yield and quality. While planting density is a key environmental factor influencing crop growth, its physiological and molecular impact on yam tuber morphogenesis remains unclear. Here, the yam ‘W48’ was used as the experimental material, and different planting density treatments were established to systematically analyze the morphological, physiological, and molecular response characteristics of yam tuber growth and development under planting density stress. Morphological analysis showed that 110 days after planting at different planting densities was the critical period for the differences in yam tuber thickness, with the tuber thickness in the low-density treatment significantly higher than in the high-density treatment. Compared with high-density treatment, the photosynthetic capacity and tuber starch content of yam plants under low-density planting were significantly improved. Cytological observation further indicated that the increase in tuber cell enlargement was the main reason for the thickening of yam. Combined transcriptomic and metabolomic analyses revealed that differentially expressed genes (DEGs) and differentially accumulated metabolites (DAMs) were mainly enriched in plant hormone signal transduction and carotenoid biosynthesis pathways. In addition, through weighted gene co-expression network analysis (WGCNA) and the expression profile analysis of key genes in hormone signaling pathways, we identified core regulatory factors, including *ARF11*, *ERF1*, and *ERF5-like*, that were significantly associated with yam tuber development. Together, this study revealed a potential mechanism by which planting density regulates yam tuber development at the morphological, physiological, and molecular levels, providing a theoretical basis for high-yield and high-quality yam cultivation and for improving tuber morphology.

## 1 Introduction

Yam (*Dioscorea opposita* Thunb.) is an annual or perennial vine plant belonging to the genus *Dioscorea* of the Dioscoreaceae family. Its tubers serve as both medicine and food (Epping and Laibach, 2020). These tubers are rich in starch and sugars, and also contain bioactive compounds such as saponins, flavonoids, and phenolic acids (Li et al., 2023). The tuber’s length, thickness, and other morphological features directly impact both quality and yield. If the yam tubers were uneven in length and thickness, it would seriously affect their commercial value and quality. Therefore, research into the mechanisms of tuber morphological development is crucial for improving the quality and efficiency of the yam industry.

The morphogenesis of tubers is influenced by genetic and environmental factors (Zierer et al., 2021). Environmental factors, including water, nutrients, light, temperature, soil mechanical resistance and structure, play an important role in influencing the morphological development of plant tubers by affecting processes such as cell division and elongation, and material distribution (Bao et al., 2025). A typical example of light affecting plant tuber formation was *SELF-PRUNING 6A* (*StSP6A*), which promotes cell division and enlargement by regulating various hormone signaling pathways (i.e., auxin, cytokinin, and gibberellin) and sugar allocation pathways, thus controlling the development of potato tubers (Bao et al., 2025; Xin et al., 1998). Additionally, studies have shown that *Phytochrome B* (*StPHYB*), *Phytochrome F* (*StPHYF*), *FD*, and *GI* (*GIGANTEA*) were also involved in regulating the tuber formation (Jackson et al., 1996; Sun et al., 2024; Valverde et al., 2004; Zhou et al., 2019). Furthermore, soil mechanical resistance, specifically soil compaction, has also been shown to affect tuber development. For instance, Zhang et al. (2026) confirmed that soil compaction stress upregulates auxin response factors in the root cortex through ethylene signaling, thereby inhibiting the expression of cellulose synthase genes. This process drives the radial expansion of cortical cells by altering cell wall, ultimately leading to thickening of the rice root. However, there were very few studies on how the environmental factors affect yam tuber morphology. Planting density was influenced the yam tuber yield and quality by altering plants photosynthetic efficiency. Nevertheless, the molecular mechanism by which planting density regulates the tuber morphology through endogenous signaling networks remains unclear.

The development of the yam tuber is usually divided into three stages: initiation, expansion, and maturation. During the expansion stage, tuber cells rapidly divide and expand. This process is accompanied by the accumulation of large amounts of storage substances such as starch and polysaccharides (Cao et al., 2023). Therefore, expansion stage is a critical period for the morphological formation of yam tubers. Some studies have shown that cytokinin (CK) and auxin (IAA) synergistically promote the initiation and expansion of storage organs in the yam tuber. In contrast, gibberellin (GA) generally exerts inhibitory effects (Cao et al., 2023; Zhou et al., 2021). Brassinosteroids (BRs) affect the shape and size of the yam tubers by regulating the expression of genes related to cell enlargement (Riekötter et al., 2023). Besides plant hormones, transcription factors (TFs) and small RNAs also play important roles in the development of yam tubers. miRNA160, miRNA396, miRNA535, and miRNA5021 have been shown to regulate cell division and differentiation during the expansion stage of yam tubers (Zhou et al., 2020).

Here, this study aims to explore how planting density affects the morphological development of yam tubers. Firstly, the morphology and structure of the tubers at different developmental stages across different planting densities were systematically evaluated using morphological and tissue sectioning techniques. Meanwhile, the photosynthetic indicators of the plants and the dynamic changes of the carbohydrate contents in the tubers were measured. Furthermore, the molecular regulatory network underlying it was systematically analyzed by integrating transcriptomic and metabolomic data. The results will provide a theoretical basis for elucidating the biological mechanism of tuber development in *Dioscorea*, and also provide important references for improving the yield and quality of characteristic yam and promoting sustainable industrial development.

## 2 Materials and methods

### 2.1 Plant materials planting and collection

The ‘W48’ yam (*D. opposita* Thunb.) was planted in the resource garden of the Institute of Chinese Herbel Medicines, Henan Academy of Agricultural Sciences in Xinxiang city, Henan Province, China (34°47′27″ N, 113°40′23″ E) in the year 2025. The plants were supported by bamboo stakes, arranged in single rows with a furrow depth of 50 cm. Two planting densities were established 50 cm × 44 cm (row spacing × plant spacing, low density) and 50 cm × 14 cm (row spacing × plant spacing, high density), with three replicate plots for each density.

### 2.2 Sample collection and phenotype analysis

The yam tubers with uniform size of without disease and undamaged were collected 90, 110, 130, and 150 days after planting (DAP), with at least three plants collected each time. After rinsing with ddH_2_O, the tubers were measured for diameter, length, and weight (Cao et al., 2023). Subsequently, all gathered samples were stored at -80 °C until use.

### 2.3 Paraffin sectioning analysis

Fresh yam tubers of the middle portion were excised with a scalpel and fixed in a solution of formaldehyde, acetic acid, and 70% ethanol solution (volume ratio 18:1:1) for 48 h. After fixation, samples were dehydrated sequentially 75%, 85%, 90%, 95%, and 100% ethanol. The samples were then cleared by immersing them, in sequence, into solutions of xylene and anhydrous ethanol mixed at 1:2, 1:1, and 2:1 (by volume), followed by pure xylene. Next, samples were infiltrated with paraffin wax at 65 °C. The tissues were embedded using the JB-P5 embedding machine (Wuhan Junjie Electronic Co., Ltd., China), and paraffin blocks were sectioned into 4 μm-thick slides using an RM2016 microtome (Shanghai Leica Instrument Co., Ltd., China). The sections were then dewaxed, stained with safranin O and fast green, and sealed. Finally, the stained sections were observed using a Nikon Eclipse E100 upright optical microscope (Nikon, Japan).

### 2.4 Measurement of plant photosynthetic rate and sucrose and starch content in tubers

The net photosynthetic rate of the yam plants was measured using a portable photosynthesis system (LCpro T Advanced Portable Photosynthesis System ADC, Inc., Lincoln, UK), with measurements taken from 8:30 am to 11:00 am. To ensure the repeatability and accuracy of physiological data, we selected 10 leaves from different directions of the upper, middle, and lower parts of the yam plant to measure the photosynthetic rate. According to the method of Dai et al. (2009), the content of chlorophyll a, chlorophyll b, and carotenoids in yam leaves was determined. Chlorophyll was extracted with 80% acetone, and the sample was treated in the dark for 24 h. The absorbance was measured using a spectrophotometer (UV-1000, Aoe Instrument Co., LTD, China) at wavelengths of 470, 649, and 665 nm. Five biological replicates were set for each treatment.

The starch content in the yam tubers was determined using the starch content assay kit (AKSU015M, Boxbio, China). 50 mg of the sample was weighed and ground into powder in a mortar and pestle. 1 mL elution solution was added and thoroughly mixed. The sample was then treated at 80 °C in a water bath for 30 min, followed by centrifugation at 8000 g for 10 min, and the precipitate was retained. 500 μL of distilled water was added, and the mixture was mixed well. The sample was treated at 95 °C in a water bath for 15 min. The samples were cooled to room temperature (RT, 25 °C). 1 mL of the extraction solution was added and the sample was extracted at RT for 15 min. The sample was centrifuged at 8000 g for 10 min, and the supernatant was collected. The absorbance of the supernatant was measured at 620 nm using a multimode microplate reader (Varioskan ALF, Thermo Fisher Scientific, USA). A standard curve was established according to the instructions, and the starch content in the yam tubers was calculated.

### 2.5 RNA-seq data analysis

Total RNA was extracted from the yam tubers using the CTAB method (Allen et al., 2006), with three biological replicates for each sample. RNA quality was evaluated using a Qubit 4.0 fluorometer (Qubit 4.0, Thermo Fisher Scientific, USA) and a Qsep400 high-throughput nucleic acid and protein analysis system (QSEP400, Guangding Biotech, China). Qualified RNA samples were used to isolate mRNA, which was then used to synthesize cDNA. Subsequent steps included end repair, dA-tailing, and sequencing adapter ligation to generate a library with 250–350 bp insert fragments. The library further purified, PCR amplification and subjected single-stranded cyclization. The final library was amplified with phi29 polymerase to prepare DNA nanoballs (DNB) containing over 300 copies of each molecule. The DNBs were loaded into the sequencing chip and sequenced on the MGI sequencing platform.

The raw data obtained from the adapters were processed through base calling and Bcl2Fastq conversion to yield raw reads. After filtering the data with fastp (Chen et al., 2018), clean reads were obtained. The clean reads were aligned to the reference genome using HISAT2 (Kim et al., 2015) and StringTie (Pertea et al., 2015), with default parameters employed for the alignment (**Supplementary Table 1**). The gene alignment results were calculated using featureCounts, and the Fragments Per Kilobase Million (FPKM) for each gene were determined based on gene length. To determine differentially expressed genes (DEGs) between samples, DESeq2 was used to analyze between two groups (Love et al., 2020; Varet et al., 2016), with genes having an FDR < 0.05 and |log_2_FC| ≥ 1 considered DEGs. Enrichment analysis was conducted based on the hypergeometric test, and for Kyoto Encyclopedia of Genes and Genomes (KEGG, https://www.genome.jp/kegg), the hypergeometric distribution test was performed at the pathway level.

### 2.6 Weighted gene co-expression network analysis and data analysis

To further identify the genes related to yam tuber development, a weighted correlation network analysis (WGCNA) was performed on 18 samples using the R package (Langfelder and Horvath, 2008). The gene co-expression network was constructed using the blockwiseModules function with the following parameters: minModuleSize = 50, powerEstimate = 18, mergeCutHeight = 0.25 (i.e., merging within modules with feature gene correlation greater than 0.78). Furthermore, hub genes were selected using the chooseTopHubInEachModule function of WGCNA. Genes with gene significance (GS) ≥ 0.8, module membership (MM), and weighted correlation index ≥ 0.8 were defined as hub genes strongly associated with yam tuber growth.

### 2.7 Metabolomic analysis

Each individual yam tuber was placed in a lyophilizer (Scientz-100F, Scientz Freeze Drying, China) under vacuum for 63 h, then ground into powder using a grinding instrument (MM 400, Retsch, Germany) at 30 Hz and for 1.5 min. The extraction process was performed using the previously reported method (Liu et al., 2024). Weigh 30 mg of sample powder using an electronic balance and add 1.5 mL of -20 °C pre-cooled 70% methanolic aqueous internal standard extract. The mixture was vortexed once every 30 min for 30 s, a total of 6 times. After centrifuging at 12000 rpm for 3 min, the supernatant was extracted and the samples were filtered through a 0.22 μm microporous membrane. The solutions were collected and stored in the injection vial for subsequent UPLC-MS/MS analysis.

All samples were collected using an LC-MS system according to the machine orders. The analysis conditions were as follows: ACQUITY UPLC HSS T3 Column (2.1 mm × 100 mm, 1.8 µm; Waters, USA). The mobile phase A was composed of an aqueous solution containing 0.1% formic acid, and the mobile phase B was composed of acetonitrile and 0.1% formic acid. The column temperature was maintained at 40 °C. The flow rate was set at 0.40 mL/min. The injection volume was 4 μL. Sample measurements were performed using a gradient program with initial conditions of 95% A and 5% B. The gradient program was as follows: 5 min (35% A and 65% B), 6 min (1% A and 99% B), 7.5 min (1% A and 99% B), 7.6 min (95% A and 5% B), 10 min (95% A and 5% B).

Mass spectrometry detection was performed in positive and negative ion scanning modes, with a positive ion volage of 5000 V and a negative ion voltage of -4000 V. Other parameters were described as follow: ion source gas 1 (GAS1), 50 psi; ion source gas 2 (GAS2), 60 psi; curtain gas (CUR), 35 psi; temperature (TEM), 550 °C, or 550 °C; declustering potential (DP), 80 V, or -80 V in positive or negative modes, respectively; and ion spray voltagefloating (ISVF).

The raw data obtained from the mass spectrometer were converted to mzML format by ProteoWizard, and peak extraction, alignment, and retention time correction were performed using the XCMS program (Alseekh et al., 2021; Li et al., 2024). Peaks with a missing rate of > 50% in each group were filtered, and blank values were filled by KNN + 1/5 minimum value (fill the blank values > 50% with 1/5 minimum value, and fill the blank values < 50% with KNN). The peak areas were corrected by the SVR method. Metabolite identification was carried out by integrating public and prediction databases, as was as metDNA methods. Finally, substances with a comprehensive score of 0.5 or above and a coefficient of variation (CV) value of quality control (QC) samples less than 0.5 were extracted, and the positive and negative modes were combined. Principal component analysis (PCA) and orthogonal partial least squares-discriminant analysis (PLS-DA) were performed using the base package and metaboAnalystR of R language (Eriksson et al., 2006; Thévenot et al., 2015). Differentially accumulated metabolites (DAMs) were identified using a threshold of variable importance in projection (VIP) > 1 and *p*-value < 0.05.

### 2.8 Integrative analysis of transcriptome and metabolome

Correlation analysis of metabolome and transcriptome data was conducted using Majorbio Cloud (Ren et al., 2022). The correlation coefficients between metabolites and genes in the differential groups were calculated by SciPy (Python) software (Version 1.0.0), and the interaction relationships were displayed using a nine-quadrant plot. KEGG software was utilized to overlay the changes in metabolites and genes onto the metabolic pathway map.

### 2.9 Quantitative real-time PCR analysis

According to the manufacturer’s protocol, cDNA was synthesized using the HiScript III 1st Strand cDNA Synthesis Kit (R312-01, Vazyme, China). The qPCR was performed on the qTOWER3G IVD instrument (Analytik Jena AG, Germany). The total reaction volume was 20 μL, including 2 μL cDNA, 0.4 μL each of forward and reverse primers (0.25 μM), 10 μL 2×NovoStart^®^SYBR qPCR SuperMix Plus (Novoprotein Scientific Co., Ltd., China), and 7.2 μL ddH_2_O. The one-step reaction procedure was as follows: 95 °C for 1 min, 40 cycles at 95 °C for 20 s, and 60 °C for 1 min, followed by melting curve analysis at 60-95 °C. The relative expression level of qRT-PCR was calculated using the 2^−ΔΔCt^ method previously described (Wang et al., 2024). The primers used are shown in **Supplementary Table 2**.

### 2.10 Data analysis

Significant analysis in this study was executed by the Tukey’s honestly significant difference, Student t-test and SPSS software, and the significance level (*P* < 0.05) applied.

## 3 Results

### 3.1 Morphological characterization of yam tuber development

**Figure 1:**
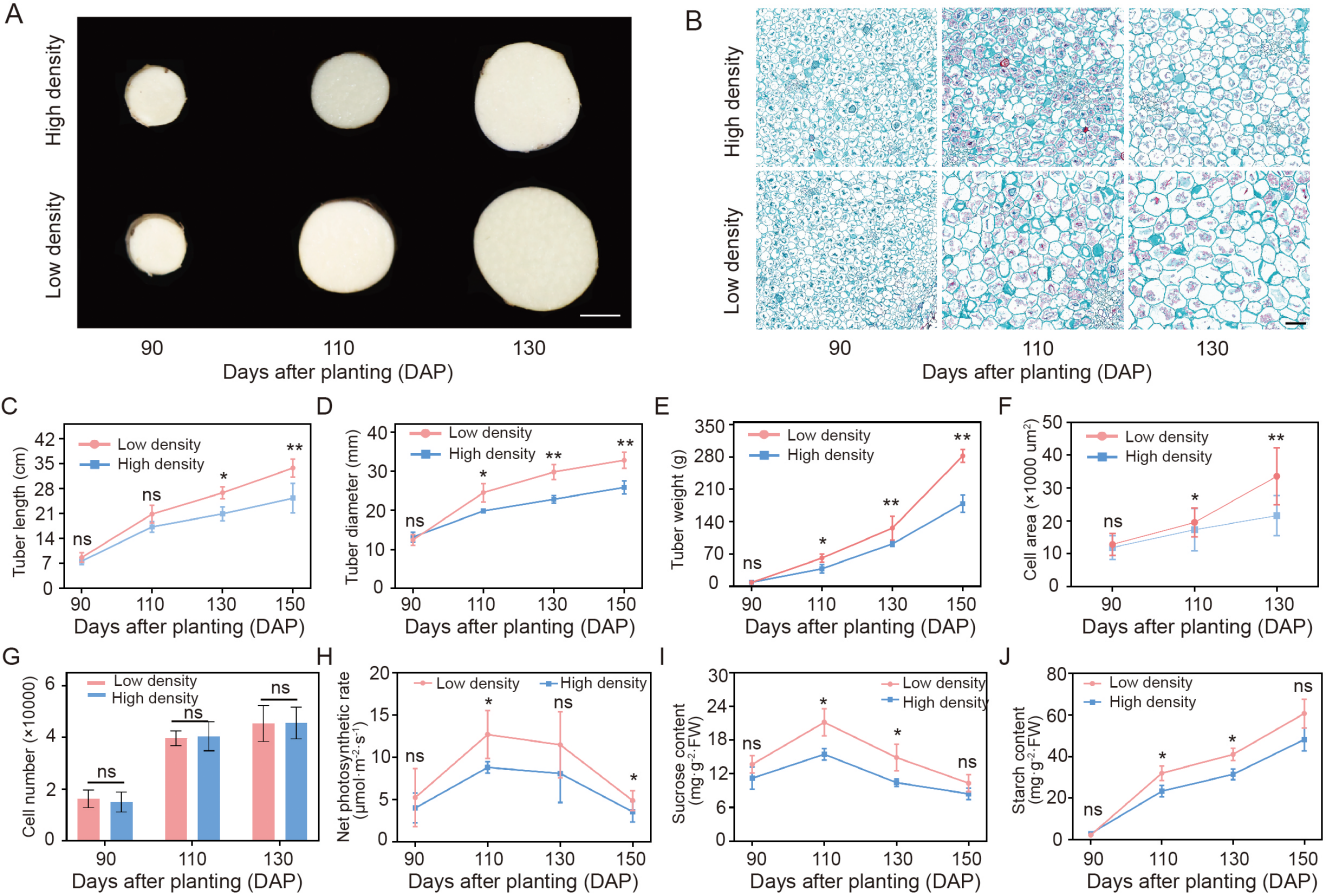
Phenotypic differences among yam tubers under different planting densities. **(A)** Morphological analysis of the cross-section of the middle part of the yam tuber at different development stages. The left side of the graph represents different planting densities. The bottom of the graph represents the DAP, days after planting. Bar = 1 cm. **(B)** The paraffin section diagram of yam tubers at the corresponding period in **(A)**. Bar = 200 μm. **(C-E)** The dynamic changes in the length, diameter, and weight of the yam tuber at different developmental stages. **(F, G)** The cell area and cell number of yam tubers at the corresponding stage in **(B)**. **(H)** The net photosynthetic rate of yam plants. **(I-J)** The dynamic changes in the sucrose and starch content of the yam tuber at different developmental stages. *p < 0.05, **p < 0.01, and ns for not significant (Tukey’s honestly significant difference test).

To elucidate the influence of planting density on yam tuber development, we collected samples at four stages of tuber development under two planting densities, and systematically measured morphology and physiology. The tuber diameters of yams under different densities showed the same trend of change. After 110 days after planting (DAP), there was a significant difference in tuber diameter between the two planting densities, with those under low- and high-densities being 25.55 ± 2.08 mm and 19.85 ± 0.45 mm, respectively (**Figures 1A, D**). Meanwhile, the observation of the yam tubers’ tissue structure of cross-section of the middle part through paraffin section showed that the cell size of yam tubers at low-density planting with 110 DAP was significantly larger than that of the high-density, while there was no significant difference in the cell number (**Figures 1B, F, G**). In addition, the yam tubers weight indicated a similar trend to the tuber diameter under two planting densities (**Figure 1E**). The tuber length showed a stable and continuous elongation trend with planting time (**Figure 1C**). However, there was a significant difference in the yam tuber length at 130 DAP, with tuber lengths being 26.87 ± 1.35 cm and 20.93 ± 1.56 cm under low- and high- density, respectively (**Figure 1C**).

Different planting densities alter plant photosynthesis, thereby affecting the accumulation of photosynthetic products and the formation of nutrient organs. Therefore, the photosynthetic rate of yam and the contents of sucrose and starch in the tubers were measured. During the growth of yam, the total chlorophyll content in the leaves showed a significant increase, followed by a decreasing trend (**Supplementary Figure 1**). The net photosynthetic rate of the plants reached its peak at 110 DAP (low-density planting was 12.70 ± 2.33 μmol·m^-2^·s^-1^, high-density planting was 8.8 ± 0.60 μmol·m^-2^·s^-1^) and then decreased. Notably, the net photosynthetic rate under low-density planting was higher than under high-density planting (**Figure 1H**). In addition, the sucrose content in the yam tubers increased from 90 DAP to 110 DAP, and then gradually decreased. At both 110 DAP and 130 DAP, sucrose content in the yam tubers was significantly higher under low-density planting (**Figure 1I**). The starch content showed an upward trend during tuber development, which was a rapid accumulation from 130 to 150 DAP (**Figure 1J**).

Altogether, we determined that 110 and 130 DAP across the two planting densities were the critical time points for the phenotypic differentiation of tuber diameter and tuber length.

### 3.2 The comparative transcriptome analysis of the yam tuber

To investigate the molecular mechanism by which planting density affects the morphological construction of yam tubers, we analyzed tuber samples at 90 DAP, 110 DAP, and 130 DAP periods using RNA-seq. Principal component analysis (PCA) and samples clustering tree analysis indicated a good consistency among the three biological replicates (**Supplementary Figures 2A, B**). Furthermore, to further validate the reliability of RNA-seq data, we randomly selected 9 DEGs for qRT-PCR. The results showed that RNA-seq and qRT-PCR results were consistent, further demonstrating the reliability of the RNA-seq results (**Supplementary Figure 3**).

**Figure 2:**
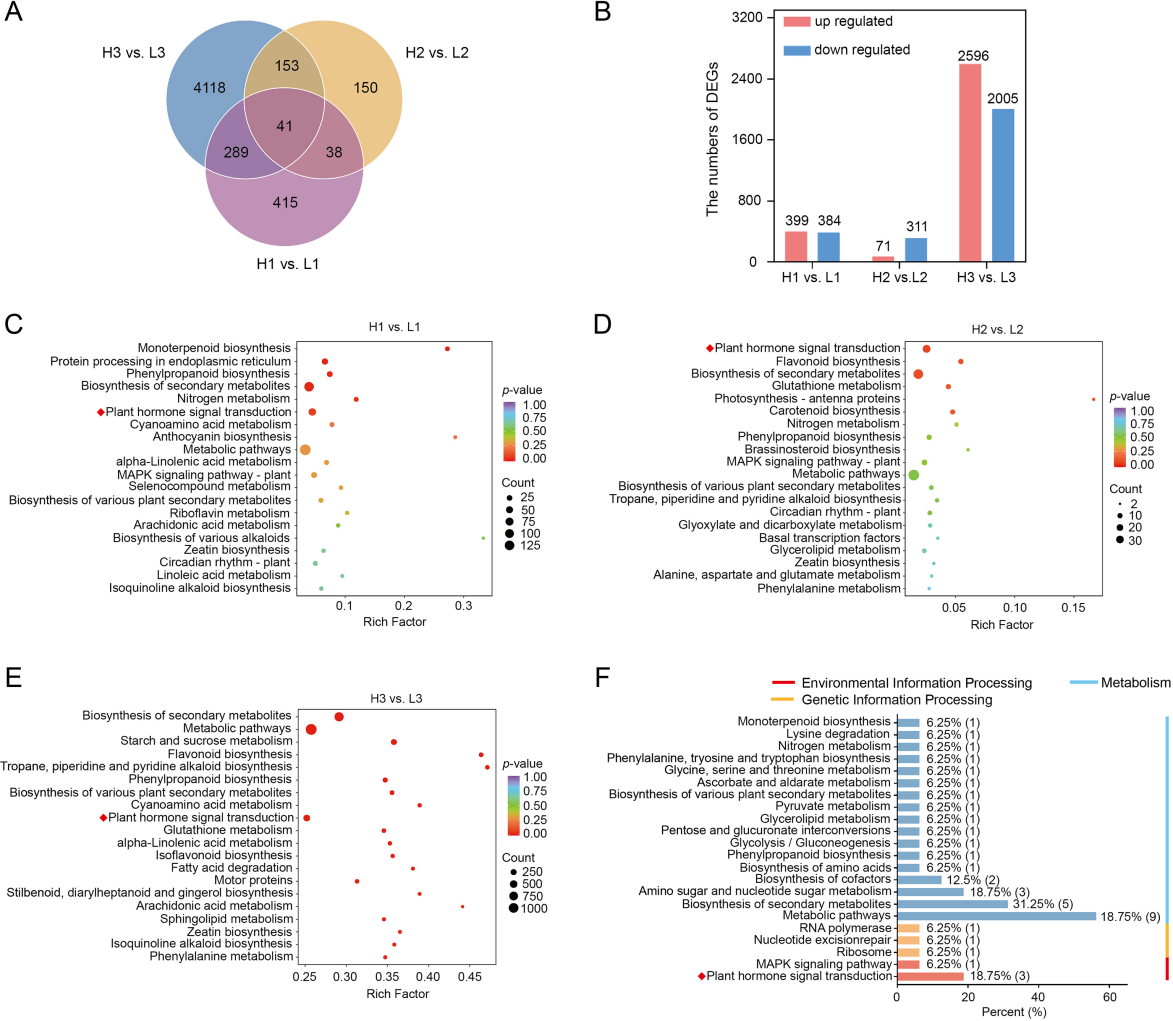
Transcriptome analysis of the yam tuber. **(A)** Venn diagram of DEGs between different planting densities. **(B)** Numbers of DEGs in the pair-wise comparisons. **(C-E)** Top 20 pathways enriched in KEGG across the three comparison groups (H1 vs L1, H2 vs L2, and H3 vs L3). **(F)** KEGG enrichment analysis of the 41 common DEGs in the three comparison groups. L and H represent planting yam at low- and high-density, respectively. The numbers following the plant density indicate the sampling stages: 1 for 90 DAP, 2 for 110 DAP, and 3 for 120 DAP.

Subsequently, we compared the transcriptome of yam tubers between different planting densities during the same period to determine how planting density affects the gene expression abundance of the yam tubers. 399, 71, and 2596 genes were identified as upregulated in the H1 vs L1, H2 vs L2, and H3 vs L3 comparison, respectively (**Figures 2A, B**). Similarly, 384, 311, and 2005 genes were downregulated in the same three comparisons, respectively (**Figures 2A, B**). The Venn diagram showed that 41 common DEGs were identified (**Figure 2A**; **Supplementary Table 3**). To further interpret these results, we performed KEGG enrichment analysis on DEGs to reveal the biological significance of these gene sets. In H1 vs L1, there are six significantly enrichment pathways, mainly including biosynthesis of secondary metabolites, protein processing in endoplasmic reticulum, and plant hormone signal transduction (**Figure 2C**). There are six significantly enrichment pathways in H2 vs L2, such as plant hormone signal transduction, flavonoid biosynthesis, and biosynthesis of secondary metabolites (**Figure 2D**). In H3 vs L3, KEGG enrichment analysis indicated that the main enrichment was in biosynthesis of secondary metabolites, metabolic pathways, starch and sucrose metabolism, and plant hormone signal transduction (**Figure 2E**). In addition, KEGG enrichment analysis of 41 common DEGs in three comparisons showed that they were mainly enriched in carbohydrate and energy metabolism, biosynthesis of secondary metabolites, and plant hormone signal transduction (**Figure 2F**). Further, we also analyzed the transcription factors. We also identified 88, 40, and 342 transcription factors in H1 vs L1, H2 vs L2, and H3 vs L3, mainly belonging to the AP2/ERF and MYB families (**Supplementary Figures 2C-E**; **Supplementary Tables 4**-**6**). Notably, several homologous genes associated with root development in other plants were found in 41 common genes, such as *EXB16*, *EXPA8*, *BRH1*, *MYB30*, and *PYL2* (**Supplementary Table 3**; Fàbregas et al., 2025; Sakaoka et al., 2018).

It is evident that the influence of planting density on the morphological construction of yam tubers mainly involves biosynthesis of secondary metabolites and plant hormone signal transduction.

### 3.3 Weighted gene co-expression network analysis

**Figure 3:**
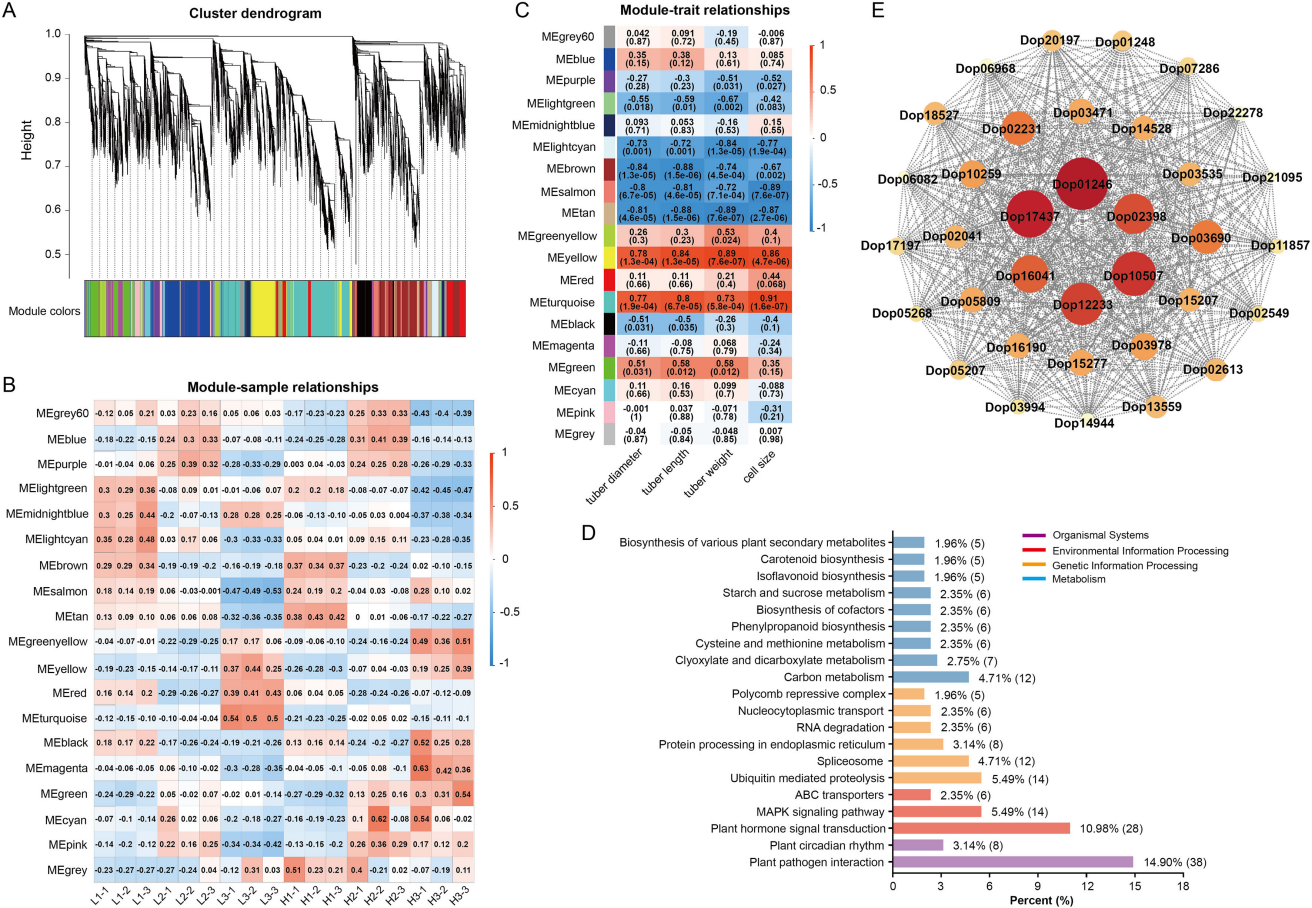
WGCNA analysis of yam tuber development transcriptome. **(A)** Clustering dendrogram of all genes, the color rows provide a simple visual comparison of module assignments based on the dynamic hybrid branch cutting method. **(B)** Heatmap showed the correlation between samples (bottom panel) and modules (right panel). Red represents positive correlation, while blue represents a negative correlation, the intensity of the color reflects the strength of the correlation. **(C)** Heatmap showing the weighted correlations between 19 modules (left panel) and 4 phenotypic traits (bottom panel). **(D)** KEGG pathways enrichment analysis of genes highly correlated with the yellow and turquoise modules. **(E)** The co-expression network of DEGs in the MEturquoise module.

Next, weighted gene co-expression network analysis (WGCNA) identified the gene modules and key genes that might be affected by planting density in the length and diameter of the yam tuber (**Figure 3**). All genes in 18 samples were used in the WGCNA analysis, and dynamic tree cutting and module merging identified 19 distinct gene modules (**Figures 3A, B**). Among them, the MEyellow (r^2^ = 0.84, *p* = 1.3e-0.5) showed the highest correlation with tuber length (**Figure 3C**), indicating that the genes within this module were highly responsive to yam tuber length. Meanwhile, MEturquoise (tuber diameter: r^2^ = 0.77, *p* = 1.3e-04; cell size: r^2^ = 0.91, *p* = 1.6e-07) had the highest correlation with the tuber diameter and cell size of tuber cross-section (**Figure 3C**), which also reflected that the genes within this module were significantly positive correlation with the tuber diameter and cell size of tuber. Additionally, the scatterplots of module membership (MM) versus gene significance (GS) further confirmed their association with the length and diameter of the yam tuber (**Supplementary Figure 4**). Collectively, the genes within MEyellow and MEturquoise modules exhibited the strongest correlation with the development of yam tubers.

Subsequently, to identify hub genes, 343 and 306 genes were identified in MEyellow and MEturquoise, each with MM ≥ 0.8 and GS ≥ 0.8 (**Supplementary Tables S7**, **S8**). To further understand their roles, KEGG analysis indicated that the genes in MEyellow and MEturquoise modules were mainly enriched in carbon metabolism and plant hormone signal transduction pathways (**Figure 3D**), respectively. It is worth noting that among the core genes selected by the MEyellow module, there are 20 TFs, such as WRKY20 (Dop04156), SHI/STY (Short internodes/stylish, Dop07700), and ERF1-like (Ethylene response factor 1, Dop09301) (**Supplementary Table 7**). Similarly, in the MEturquoise module, 23 TFs, including ARF11 (Auxin response factor 11, Dop02398), RHL1 (Root hairless 1, Dop21180), bHLH7-like (Dop18414), NF-YB3 (Nuclear transcription factor Y subunit beta, Dop03606), were identified among the hub genes (**Figure 3E**; **Supplementary Table 8**).

### 3.4 Comparative analysis of metabolomics in yam tuber

**Figure 4:**
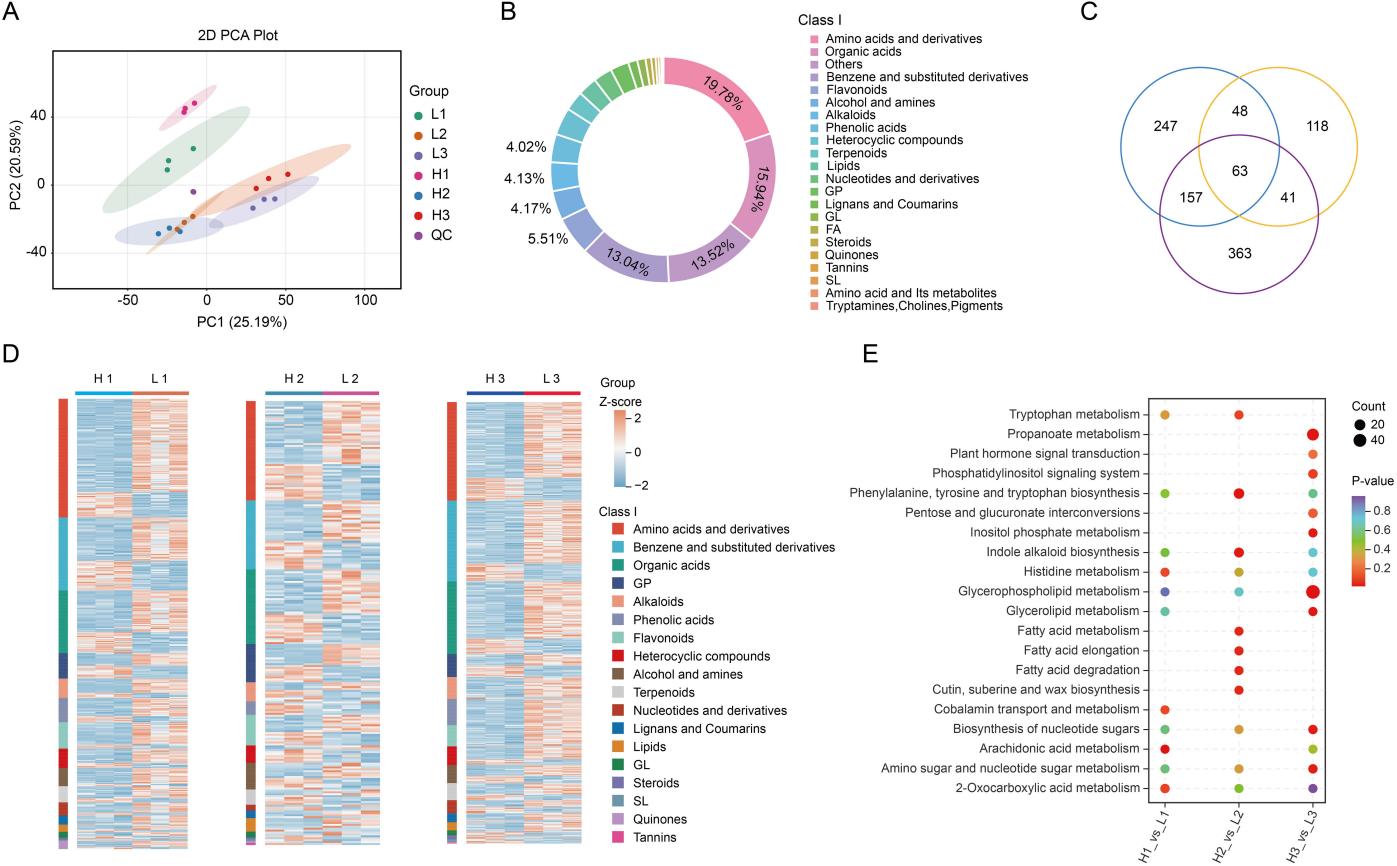
Metabolome analysis of yam tuber under different planting densities. **(A)** 18 metabolome samples were subjected to PCA, principal component analysis. **(B)** Classification of the metabolites of yam tuber samples. **(C)** Venn diagram of DAMs between H1 vs L1, H2 vs L2, and H3 vs L3. In total, 63 overlapping DAMs were identified. **(D)** Heatmap showing DAMs clustering analysis for each treatment group in the yam tuber. **(E)** The top 20 enriched KEGG pathways across the three comparison groups.

We further analyzed changes in metabolite abundance in yam tubers under two planting densities using metabolomics. Principal component analysis (PCA) and a sample cluster dendrogram were performed, and good consistency was observed among the three biological replicates (**Figure 4A**; **Supplementary Figure 5A**). PC2 could explain 20.59% of the variation and could distinguish samples from the 110 DAP from those in the 90 DAP, while PC1 (25.19% of the total variation) could distinguish samples from the 120 DAP from those of the other two stages (**Figure 4A**). Additionally, *Pearson* correlation coefficients were used to analyze the correlations between different samples, further indicating that the metabolome data had good repeatability and high data reliability (**Supplementary Figure 5B**).

Through metabolomics analysis of 18 samples, a total of 22 class metabolites were identified, with amino acids and derivatives accounting for the highest proportion of 19.78%, organic acids accounting for 15.94%, and benzene and substituted derivatives accounting for 13.04% (**Figure 4B**). To further investigate the effect of planting densities on the metabolites of yam tuber, we compared the DAMs of the tuber in different planting densities during the same stages. Specifically, 146, 123, and 171 up-regulated DAMs were identified in H1 vs L1, H2 vs L2, and H3 vs L3, respectively (**Figure 4C**; **Supplementary Figure 5C**). In parallel, 369, 147, and 453 down-regulated DAMs were detected in the three group comparisons, respectively (**Figure 4C**; **Supplementary Figure 5C**). In addition, the Venn results showed that 63 overlapping DAMs were identified across the three comparison combinations (**Figure 4C**; **Supplementary Figure 5**; **Supplementary Table 9**). It is worth noting that among the three comparison combinations, amino acids and derivatives, benzene and substituted derivatives, and organic acids have the highest proportion of differential metabolites (**Figure 4D**). Following the identification of DAMs, we performed KEGG pathway enrichment analysis. In the H1 vs L1 comparison, the main pathways included arachidonic acid metabolism, 2-oxocarboxylic acid metabolism, cobalamin transport and metabolism, and Histidine metabolism (**Figure 4E**). In the comparison between H2 and L2, DAMs were predominantly enriched in phenylalanine, tyrosine and tryptophan biosynthesis, indole alkaloid biosynthesis, and the tryptophan metabolism pathway (**Figure 4E**). The enriched KEGG pathways primarily involved propionate metabolism, glycerophospholipid metabolism, and amino sugar and nucleic sugar metabolism in the comparison of H3 with L3 (**Figure 4E**).

### 3.5 Integrative analysis of transcriptome and metabolome

**Figure 5:**
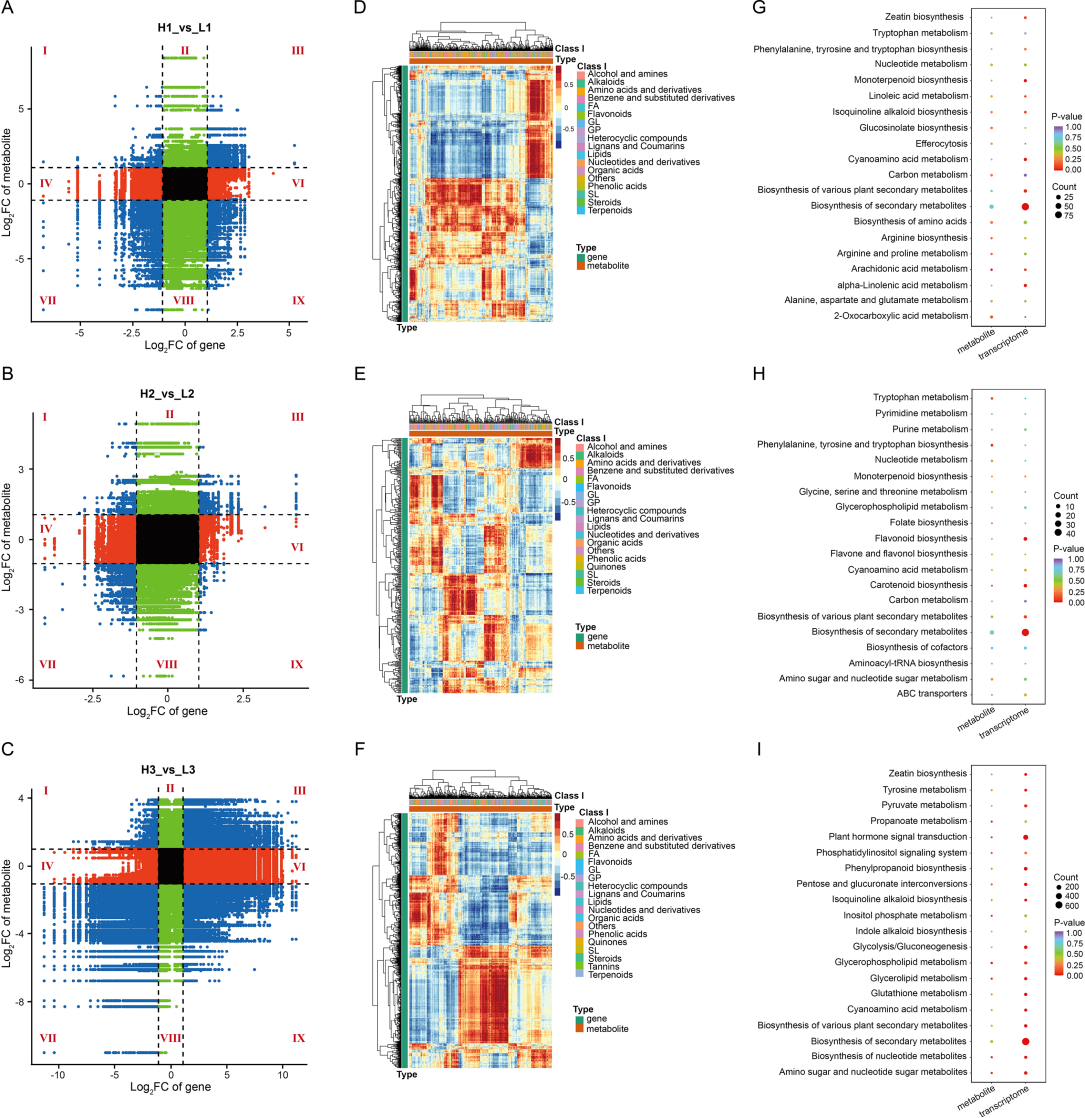
Overall information on the combined analysis of transcriptomics and metabolomics of the yam tuber. **(A-C)** shows correlation analysis of transcriptome and metabolome in H1 vs L1, H2 vs L2, and H3 vs L3, respectively. I and IX, opposite change trend in DEGs and DAMs; III and VII, the same change trend in DEGs and DAMs; II, IV, VI and VIII, non-correlation in DEGs and DAMs. **(D-F)** presents correlation coefficient clustering of the associated DEGs and DAMs in H1 vs L1, H2 vs L2, and H3 vs L3, respectively. **(G-I)** illustrates KEGG pathway enrichment bubble plot in H1 vs L1, H2 vs L2, and H3 vs L3.

To better understand the relationship between the transcriptional levels and metabolic product changes in yam tubers, we conducted an integrated analysis of DEGs and DAMs (**Figure 5**). Our findings revealed that there are more DEGs and DAMs exhibiting the same change trend in the comparison between H1 vs L1, H2 vs L2, and H3 vs L3 (**Figures 5A–C**). Furthermore, the DEGs and DAMs with consistent change trends are mainly amino acids and derivatives, benzene and substituted derivatives, and organic acids (**Figures 5D–F**). Subsequently, we conducted a combined analysis of the pathways that were significantly enriched in both the gene set (*p* < 0.05) and the metabolic set (*p* < 0.05), and produced KEGG pathway enrichment maps. For H1 vs L1, only the arachidonic acid metabolism pathway was significantly enriched (**Figure 5G**). In H2 vs L2, carotenoid biosynthesis was significantly enriched (**Figure 5H**). We identified six pathways enriched in both H3 and L3, primarily including amino sugar and nucleotide sugar metabolites, plant hormone signal transduction, pentose and glucuronate interconversion (**Figure 5I**). It is evident that at the transcriptome and metabolome levels, the planting densities mainly affects the development of yam tuber through pathways related to plant hormones and energy metabolism.

### 3.6 Changes in phytohormones during yam tuber development

**Figure 6:**
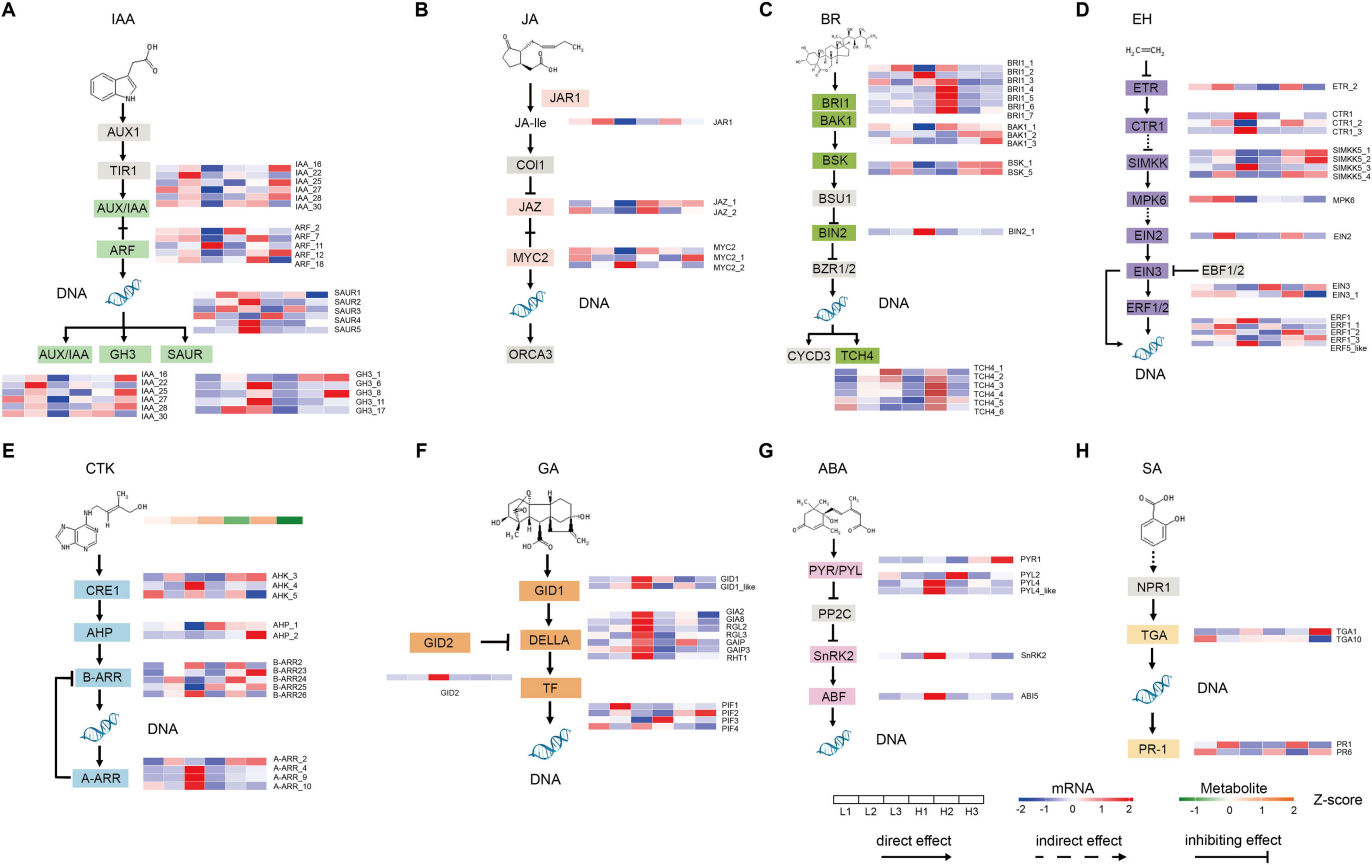
The planting densities induced changes in plant hormone signaling pathway genes and metabolite expression profiles. **(A)** Auxin, **(B)** Jasmonic acid, **(C)** Brassinosteroid, **(D)** Ethylene, **(E)** Cytokinine, **(F)** Gibberellin, **(G)** Abscisic acid, and **(H)** Salicylic acid in yam tubers at different development stages.

The above results indicated that the development of yam tubers was mainly affected by the plant hormone signal transduction pathway under two planting densities. Therefore, we focused on analyzing the expression profiles of key regulatory genes involved in plant hormone signaling (**Figure 6**). We first examined the auxin signal transduction pathway, where 21 DEGs were identified in H1 vs L1, H2 vs L2, and H3 vs L3, including 6 *IAA* genes, 5 *ARF* genes, 5 *GH3* genes, and 5 *SAUR* genes (**Figure 6A**). Under low-density planting conditions, the expression of *ARF11*, *SAUR2*, *SAUR4*, *SAUR5*, *GH3_6*, *GH3_11*, and *GH3_17* genes in yam tubers was promoted. Next, the jasmonic acid signaling transduction pathway was analyzed, where 6 genes encoding *JAR1*, *JAZ*, and *MYC2* were identified (**Figure 6B**). Similarly, 19 genes involving *BRI1*, *BAK1*, *BSK*, *BIN2*, and *TCH4* were identified in the brassinolide (BR) signal transduction pathway (**Figure 6C**). In the ethylene signal transduction pathway, 17 genes encoding *ETR* (1), *CTR* (3), *SIMKK5* (4), *MPK6* (1), *EIN2* (1), *EIN3* (2), and *ERF* (5) were identified (**Figure 6D**). 3 *AHK* genes, 2 *AHP* genes, 5 *B-ARR* genes, and 4 *A-ARR* genes were detected in the cytokinin (CTK) signal transduction pathway (**Figure 6E**). 3 *A-ARR* genes were highly expressed at L3. Interestingly, trans-zeatin continuously increased at L1-L3, while the metabolite content of trans-zeatin in H3 was the lowest. In the GA signal transduction pathway, 2 *GID1* genes, 1 *GID2* gene, 7 *DELLA* genes, and 4 *PIF* genes were detected. *GID1*, *GID2*, and *DELLA* had the highest expression levels at L3 (**Figure 6F**). In the ABA signaling pathway, the results showed that low-density planting conditions induced higher gene expression of *PYL4*, *PYL4_like*, *SnRK2*, and *ABI5* in yam tubers (**Figure 6G**). In addition, only 4 genes involving *TGA* (2) and *PR-1* (2) were identified in the SA signal transduction pathway (**Figure 6H**).

Besides, *ARF11*, *ERF1*, and *ERF5-like* genes were also key hub genes involved in the development of yam tubers. This further indicated that these genes play a crucial role in the development of yam tubers.

## 4 Discussion

Yam is an important tuber plant, and the morphology of its tuber directly determines the yield and quality (Cao et al., 2023). For example, uneven tuber lengths and thicknesses seriously affect the commercial value of yams. Tuber formation is a complex process regulated by genotype and environmental factors, involving multiple regulatory factors such as transcription factors, enzymes, and endogenous hormones (Aksenova et al., 2012). Although the regulatory mechanisms of tuber morphogenesis have been widely studied in some plants (especially model plants) (Rademacher et al., 2011; Xue et al., 2024; Zhou et al., 2019), few studies have explored how external factors affect yam tuber formation and its molecular regulatory networks. In this study, we focused on the effects of planting density on yam tuber through morphological observation, determination of physiological and biochemical indicators, and combined with transcriptome and metabolome technologies, thereby revealing the underlying molecular regulatory network.

### 4.1 Planting density affects the tuber expansion of yam by regulating photosynthetic efficiency and carbon allocation

Planting density is important environmental regulatory factor. It can affect the canopy light environment and photosynthetic efficiency by altering plant spatial layout and leaf area distribution (Long et al., 2025). Within a certain density range, low-density conditions generally benefit leaf light uniformity and light energy utilization efficiency, thereby maintaining a higher photosynthetic rate of the plant. Our results showed that different planting densities significantly affected the photosynthetic rate of yam plants at 110 DAP, accompanied by significant differences in sucrose and starch content of the yam tubers (**Figure 1**). This suggests that changes in planting density impacted the carbon metabolic process. To further understand these effects, morphological and cytological analyses revealed that the tuber thickness of yam under low-density planting conditions was significantly higher than that under high-density treatment. This finding indicated that planting density may in fluence tuber expansion by affecting the supply and distribution of photosynthetic products. Low-density planting significantly enhanced the photosynthetic capacity of yam plants, which increases the carbon source supply to the yam tuber. Consequently, the abundant carbon source input provided higher energy levels and more metabolic substrates for tuber cells, promoting cell metabolism. According to the source-sink theory, the ability of plants to form photosynthetic products and their distribution efficiency among different organs are key factors determining the degree of development of underground storage organs (Julius et al., 2017; Yang and Tang, 2026). Previous studies in potatoes have demonstrated that photoperiod signals can precisely regulate the redistribution of photosynthetic products from leaves to tubers (Tai, 2024). Simultaneously, more carbon assimilates were allocated for the synthesis of storage substances such as starch in tuber cells, which not only increases intracellular material accumulation, but may also promote cell enlargement by altering cell osmotic pressure, leading to thickening of yam tuber. Therefore, planting densities may alter carbon allocation patterns by affecting plant photosynthetic efficiency. Ultimately, we observed that yam tubers exhibited significantly greater length and thickness under low-density planting conditions compared to high-density planting conditions.

### 4.2 The carotenoid biosynthesis pathway may be involved in the regulation of the development of yam tubers

The combined transcriptomic and metabolomic analyses in H2 vs L2 revealed the DEGs and DAMs significantly enriched in the carotenoid biosynthesis pathway (**Figure 5H**). Carotenoids are important functional metabolites in plants, playing a crucial role in light energy capture and protection during photosynthesis, as well as in the formation of plant organ colors (Dong et al., 2022; Liu et al., 2024). In addition, carotenoids were precursors substance for the synthesis of various plant hormones, including abscisic acid and strigolactones (Nisar et al., 2015). Previous studies have shown that carotenoids drive root plasticity in rice through abscisic acid and strigolactones (Shrestha et al., 2026). Based on the results of our study, it is speculated that during the development of yam tuber, the carotenoid biosynthesis pathway may participate in the elongation and division of tuber cells through hormone mediated regulation, thereby affecting the expansion of tubers.

### 4.3 Plant hormones may affect yam tuber expansion through integration of planting density signals

The plant coordinates its growth and development through diverse chemical signals (Aksenova et al., 2012; Shrestha et al., 2026), among which plant hormones are the core mediators integrating external environmental signals with internal signaling molecules. Plant hormones precisely regulate plant growth and organ development by modulating intracellular and extracellular gene expression. In this study, transcriptomic and metabolomic data of yam tuber under two planting densities indicated that a large number of DEGs and DAMs in the plant hormone signaling pathway were significantly enriched (**Figures 2**, **3**). This indicated that planting density may serve as an environmental signal, mediating yam tuber development through plant hormones. WGCNA further identified that *ARF11* gene was highly correlated with the developmental traits of yam tubers. Auxin may be involved in regulating cell division and differentiation in tubers. Auxin activates membrane H^+^-ATPase activity through the *TIR1/AFB*-*AUX/IAA*-*ARF* signaling module, promoting proton efflux and causing acidification of the cell wall environment, thereby enhancing cell water absorption capacity under the drive of cell turgor pressure, ultimately promoting cell elongation and volume increase (Cancé et al., 2022; Morffy et al., 2024; Rademacher et al., 2011; Vanneste et al., 2025; Weijers et al., 2005). We speculate that *ARF11* may be involved in regulating cell enlargement, thereby promoting the expansion of yam tubers.

In addition, *ERF1* and *ERF5-like* genes have been identified as common DEGs by comparing the transcriptome of yam tubers between different planting densities during the three periods (**Figure 2A**), suggesting that ethylene signaling may also be involved in the process of yam tuber expansion. Previous studies have shown that the signal crosstalk between plant hormones plays an important role in the growth and environmental adaptation of the underground storage organ. For example, rice transforms external mechanical stress into internal signals for cell wall instructions through a cascade regulation of ethylene-*OsARF1*-cellulose synthesis, thereby enabling the root system to adapt to the external environment (Zhang et al., 2026). Based on this, it is speculated that the development of the yam tuber is regulated synergistically by other factors and ethylene signaling. Similar environmental signal transduction mechanisms have been verified in some plants (Shrestha et al., 2026).

Recent studies have further demonstrated that plant hormones not only directly regulate cellular behavior but also participate in plant organ development by reshaping the central carbon metabolism network. For example, auxin can drive plant organ development by coordinating glycolysis and TCA cycle related metabolic fluxes (Amanda et al., 2022; Fàbregas et al., 2025). Our study found that planting density significantly affects the enrichment of carbon metabolism related differential genes and metabolites in yam tuber. The plant hormones may synergistically regulate cell growth and material storage processes by promoting the synthesis and accumulation of carbohydrates such as starch. In this way, multiple hormones could mediate the regulation of planting density on yam tuber length and thickness at various levels.

### 4.4 Transcription factors may be involved in the regulation of yam tuber expansion

Transcription factors play a core role in cell differentiation, cell proliferation, and organ development. This study used multi-omics analysis to screen for candidate transcription factors involved in yam tuber development, including *MYB30*, *WRKY20*, *SHI/STY*, *RHL1*, *bHLH7-like*, and *NF-YB3*. *IbNF-YA1* can regulate the thickening of sweet potato tubers (Xue et al., 2024). *GhMYB25* and *GhMYB109* were involved in regulating the initiation and elongation of fiber cells in cotton (MaChado et al., 2009; Pu et al., 2008). In addition, *StbHLH93* affected potato tuber expansion by regulating the conversion of proplastid to amyloplast (Yang et al., 2024). Based on these precedents, we speculated that *MYB30*, *WRKY20*, *SHI/STY*, *RHL1*, *bHLH7-like*, and *NF-YB3* genes may be involved in regulating yam tuber development, and their specific functions need to be further clarified through subsequent gene function verification.

**Figure 7:**
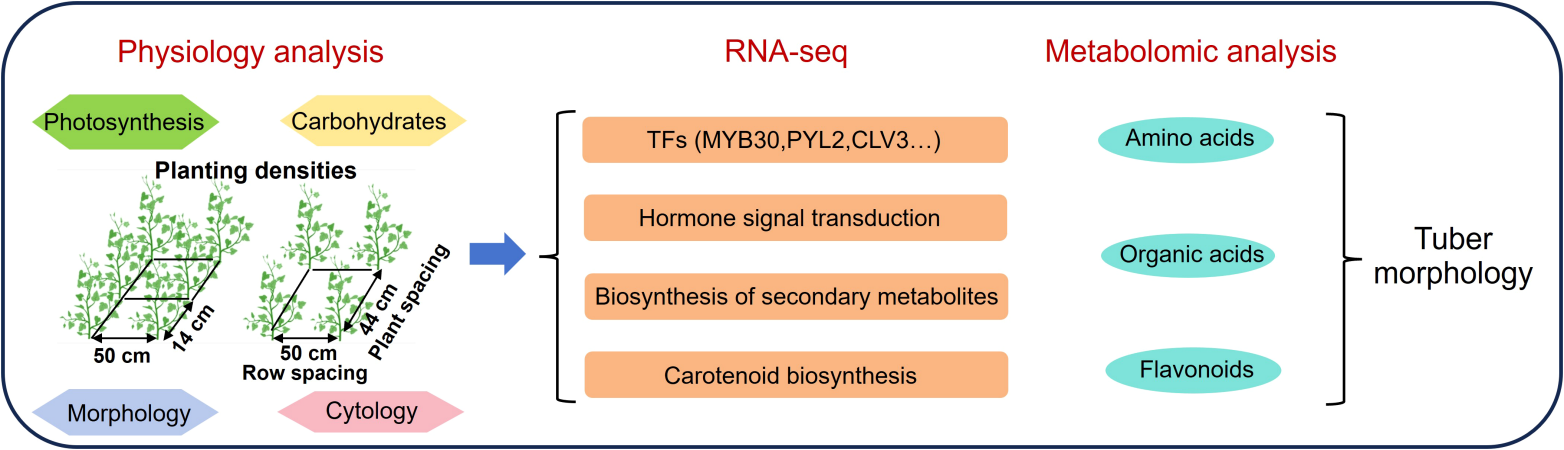
The schematic model in the regulation of yam tuber morphological formation by planting densities.

Taken together, our results systematically revealed the physiological and molecular responses of yam tuber morphological development to planting densities, providing new insights into the biological mechanisms of *Dioscorea* tuber development. A schematic model illustrating the regulation of yam tuber morphological formation by planting densities is presented in **Figure 7**.

## 5 Conclusions

This study systematically investigated the growth and physiological changes of yam tubers under two planting density conditions and revealed their potential molecular regulatory mechanisms through integrated transcriptomic and metabolomic analyses. Phenotypic data showed that 110 DAP was the critical period for yam tuber expansion, and cell enlargement was considered the main cause for the morphological differences in the tuber. Further multi omics analysis showed that the plant hormone signaling pathway and carotenoid biosynthesis pathway play a central role in yam tuber morphogenesis. Moreover, WGCNA was used to identify potential candidate genes involved in yam tuber expansion, including *ARF11*, *ERF1*, and *ERF5-like*. Overall, this study elucidated the effects of planting density on yam tuber development and metabolite accumulation, and identified core genes that may regulate tuber morphogenesis. The results not only provide a theoretical basis for understanding the development of storage organs in Dioscoreaceae plants, but also provide scientific references for optimizing yam cultivation management, improving yield and quality.

## Data Availability

The raw sequence data reported in this paper have been deposited in the Genome Sequence Archive in National Genomics Data Center, China National Center for Bioinformation/Beijing Institute of Genomics, Chinese Academy of Sciences (GSA: CRA041355) that are publicly accessible at https://ngdc.cncb.ac.cn/gsa.
