# Correction: Integrated multi-omics analysis reveals the molecular mechanism of tuber morphogenesis under different planting densities in yam (*Dioscorea opposita* Thunb.)

**DOI:** 10.3389/fpls.2026.1932431

**Published:** 2026-07-29

**Authors:** Peng Wang, Guixiao La, Xiangyang Li, Dandan Dai, Guixia Shi, Yi Wen, Tiegang Yang

**Affiliations:** 1 Institute of Chinese Herbal Medicines, Henan Academy of Agricultural Sciences, Zhengzhou, Henan, China; 2 Key Laboratory for the Protection and Utilization of Chinese Herbal Medicine Resources of Henan Province, Institute of Chinese Herbal Medicines, Henan Academy of Agricultural Sciences, Zhengzhou, Henan, China

**Keywords:** morphological formation, plant hormone signaling pathway, planting density, transcription factors (TFs), yam tubers

In the published article, there was an error in **Figure 4D**. During the assembly of the composite figure, the panel intended to represent the comparison “L1 vs H1” was mistakenly uploaded using the same image as that for “L2 vs H2”. As a result, the two panels incorrectly displayed identical images. The authors have now re-examined the original data and prepared a corrected version of **Figure 4**, in which each panel accurately reflects its corresponding comparison. The corrected **Figure 4** is provided below.

**Figure 4:**
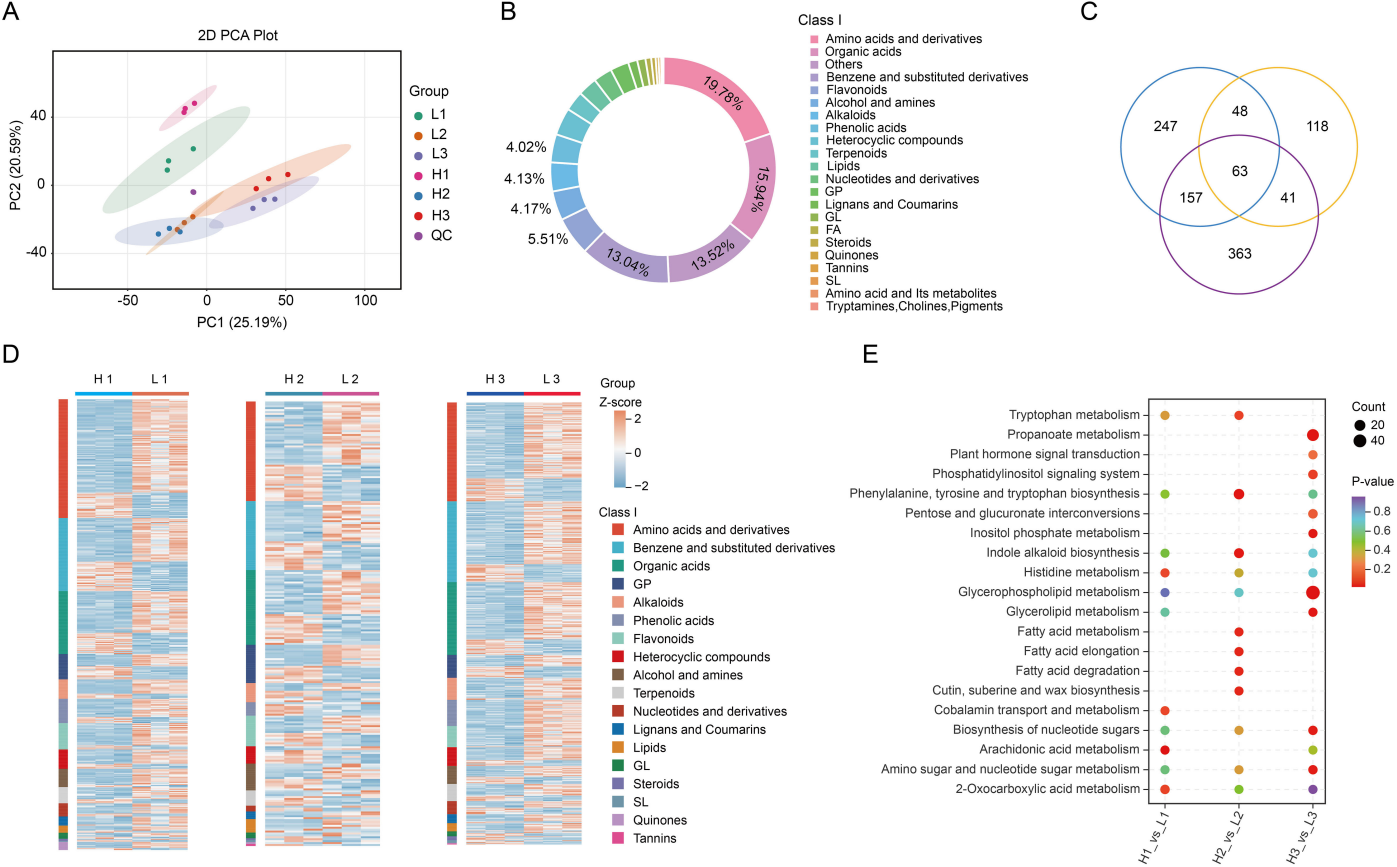
Metabolome analysis of yam tuber under different planting densities. **(A)** 18 metabolome samples were subjected to PCA, principal component analysis. **(B)** Classification of the metabolites of yam tuber samples. **(C)** Venn diagram of DAMs between H1 vs L1, H2 vs L2, and H3 vs L3. In total, 63 overlapping DAMs were identified. **(D)** Heatmap showing DAMs clustering analysis for each treatment group in the yam tuber. **(E)** The top 20 enriched KEGG pathways across the three comparison groups.

The original version of this article has been updated.

