# Supplementary material for: Integrated multi-omics analysis reveals the molecular mechanism of tuber morphogenesis under different planting densities in yam (Dioscorea opposita Thunb.)
Source: Front Plant Sci. 2026 May 19;17:1849989. doi: 10.3389/fpls.2026.1849989 (PMC13226005; doi:10.3389/fpls.2026.1849989)
Supplement: Supplementary file 1 [file DataSheet1.doc]

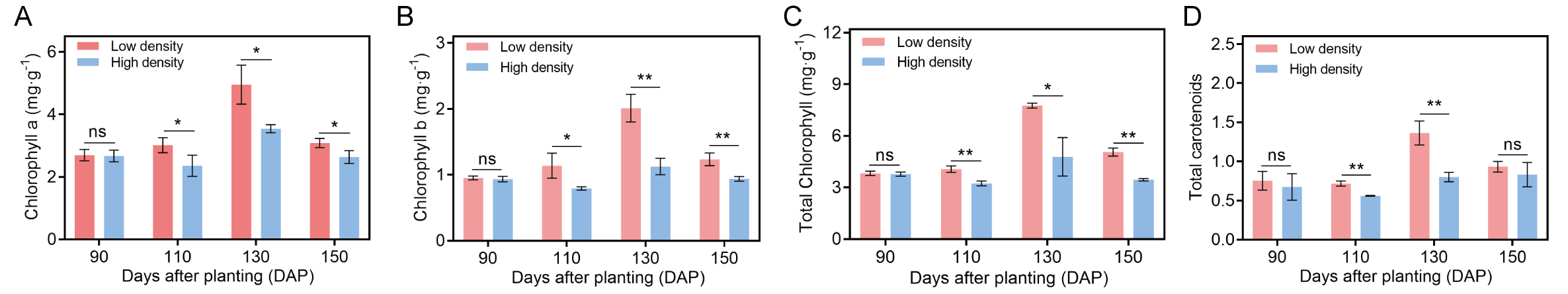


**Fig. S1** **The content of chlorophyll a, chlorophyll b, and carotenoids in yam leaves at different growth stages.** (**A**) Chlorophyll a. (**B**) Chlorophyll b. (**C**) Total chlorophyll. (**D**) Total carotenoids. **p* < 0.05, ***p* < 0.01, and ns for not significant (Tukey’s honestly significant difference test).


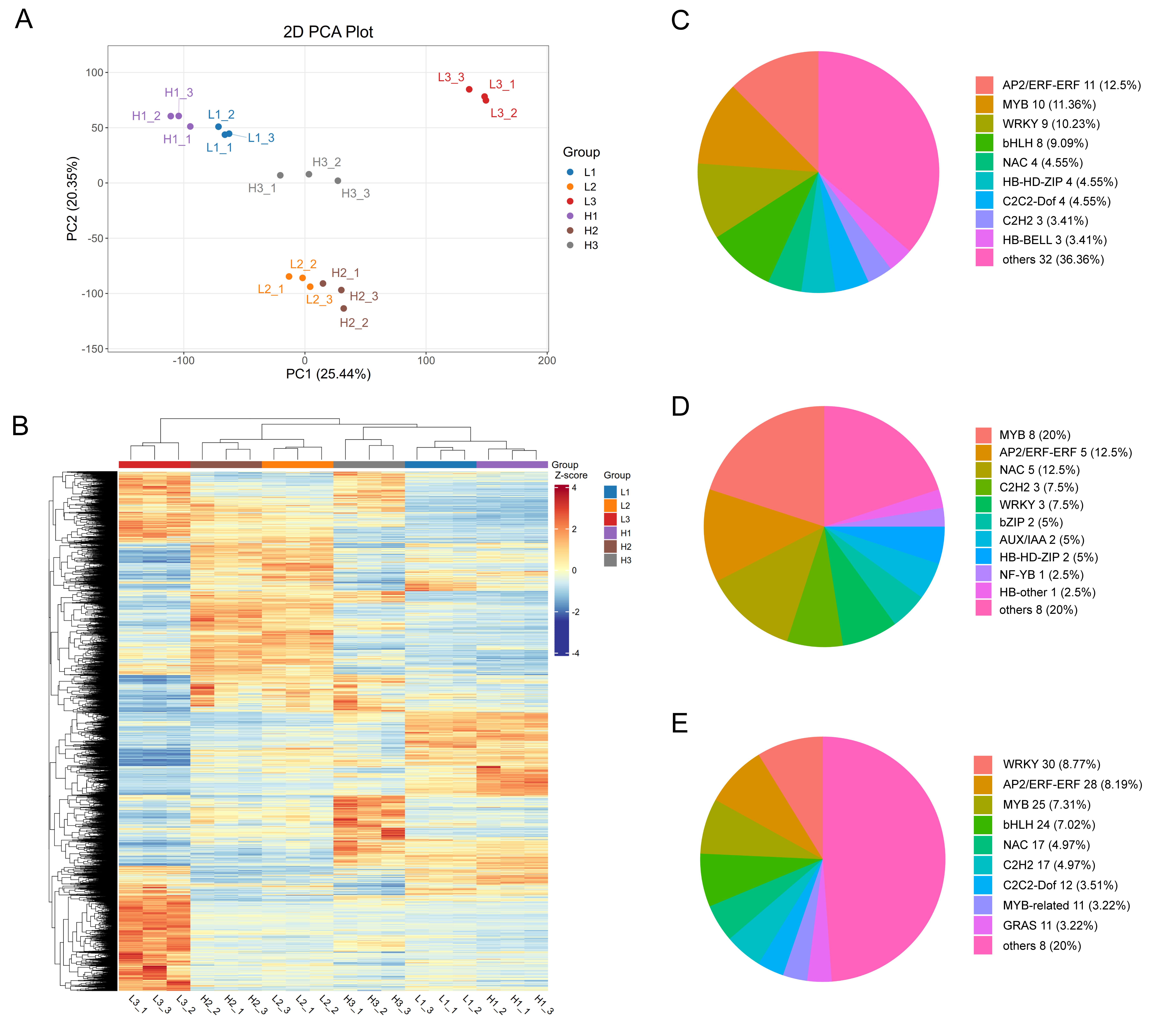


**Fig. S2 The cluster analysis of differentially expressed genes.** (**A**) Principal component analysis of transcriptome data. (**B**) The samples clustering tree analysis of transcriptome data. (**C-E**) represent the transcription factors in the DEGs in H1 vs L1, H2 vs L2, and H3 vs L3, respectively.


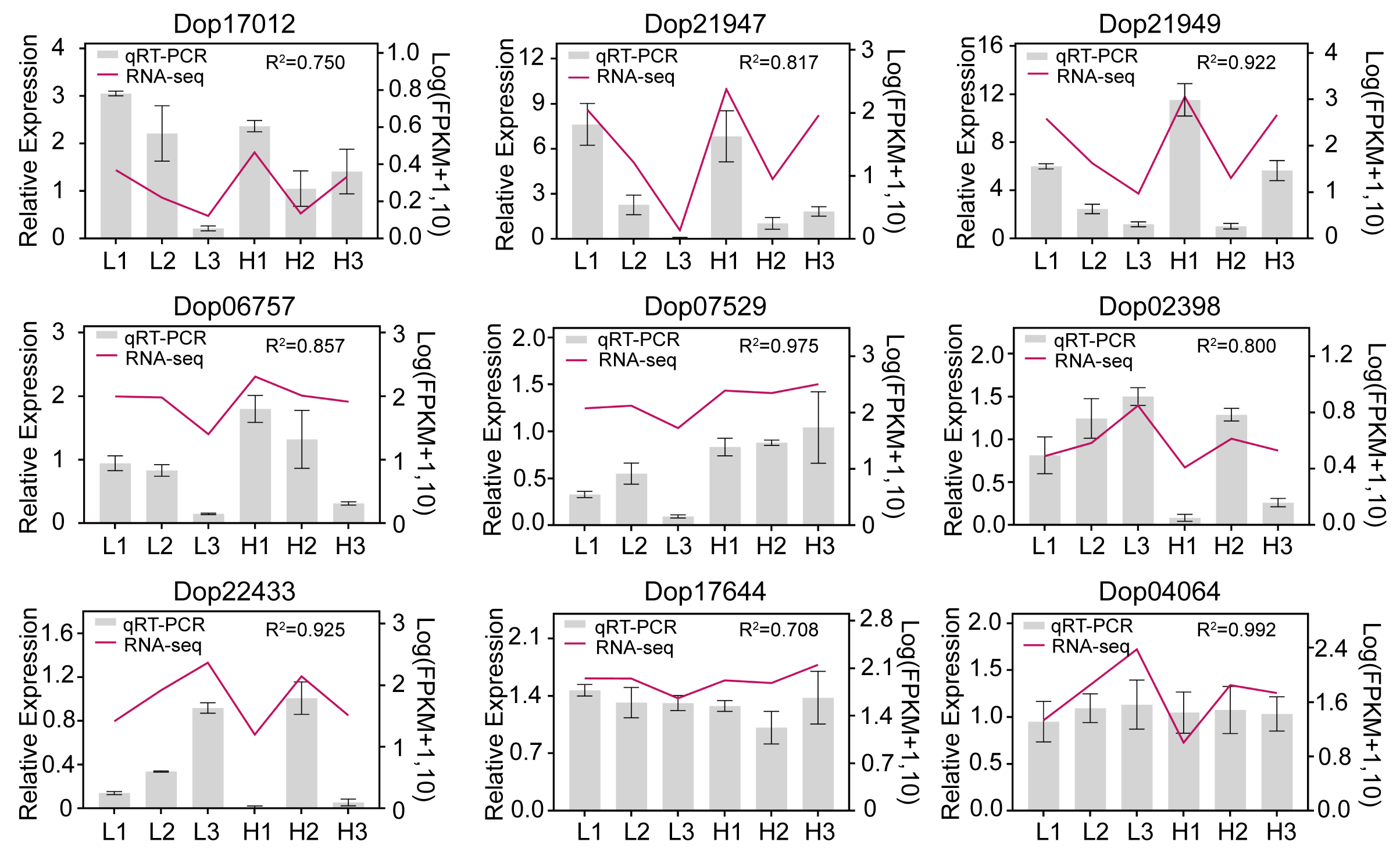


**Fig. S3 The RNA-seq data were verified via qRT-PCR**. Note: R^2^ indicated the correlation coefficient between qRT-PCR and RNA-Seq data. Line plots indicate values of log_10_(FPKM+1).


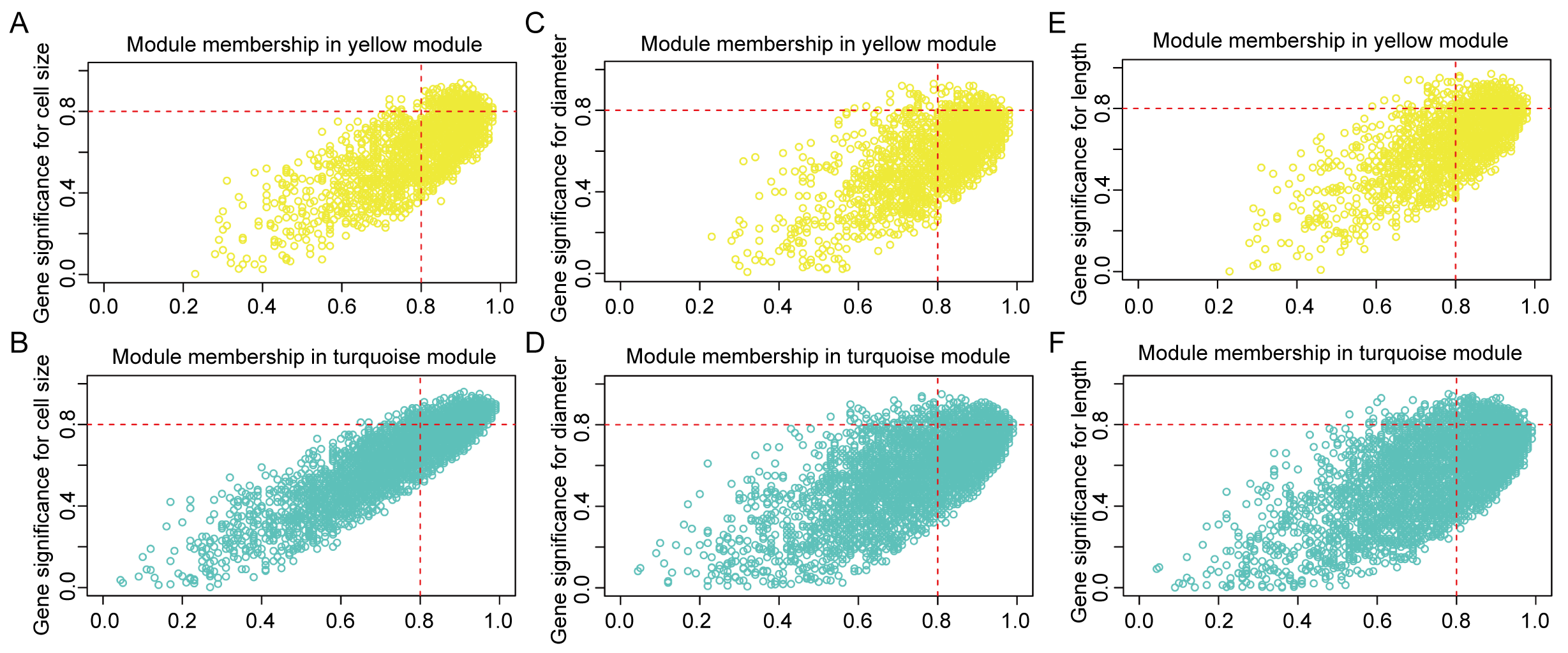


**Fig. S4** **Scatterplot showing GS for yam tuber versus MM in the yellow and turquoise modules.** (A-B) cell size. (C-D) tuber diameter. (E-F) tuber length. Each dot represents a gene.


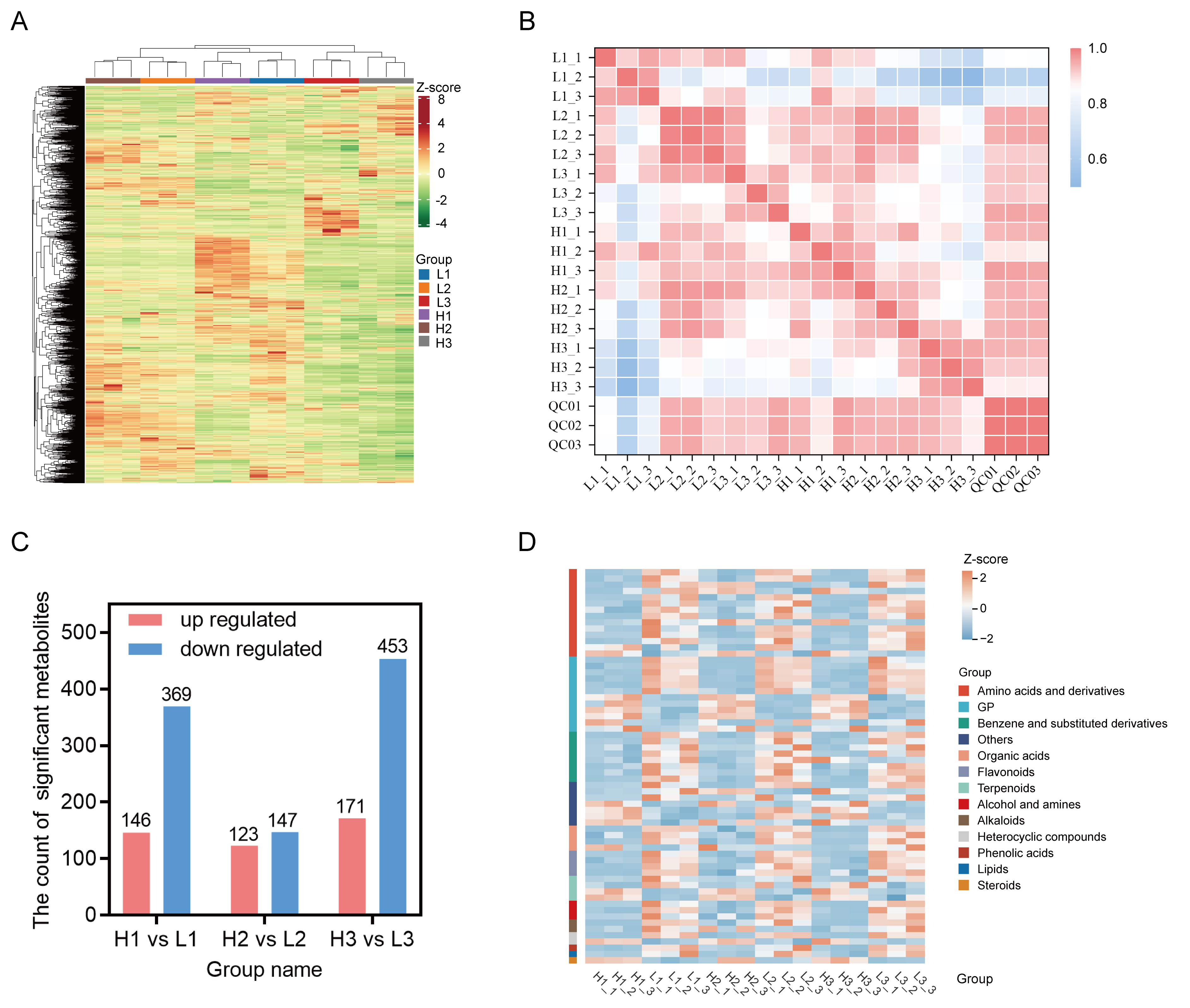


**Fig. S5 The cluster analysis of** **differential metabolites.** (**A**) Cluster analysis of differential metabolites. (**B**) Correlation analysis between samples. (**C**) The number of upregulated and downregulated differential metabolites in H1 vs L1, H2 vs L2, and H3 vs L3. (**D**) Classification of differential metabolites.
